# Efficacy of Repeated Low-Level Red Light (RLRL) therapy on myopia outcomes in children: a systematic review and meta-analysis

**DOI:** 10.1186/s12886-024-03337-5

**Published:** 2024-02-20

**Authors:** Mohamed Ashraf Youssef, Ahmed Ragab Shehata, Ahmed Moataz Adly, Mohamed Ragab Ahmed, Hoda Fahmy Abo-Bakr, Rehab Mahmoud Fawzy, Ahmed Taha Gouda

**Affiliations:** Faculty of Medicine, Beni Suef University, Beni Suef city, Beni Suef Egypt

**Keywords:** Childhood myopia, Repeated Low-Level Red Light (RLRL), Spherical equivalent refraction (SER), Axial length (AL), Sub foveal choroidal thickness (SFCT)

## Abstract

**Background:**

Myopia is the most prevalent form of refractive error that has a major negative impact on visual function and causes blurring of vision. We aimed to determine if Repeated Low-Level Red Light (RLRL) treatment is beneficial in treating childhood myopia in terms of axial length (AL), spherical equivalent refraction (SER), and sub foveal choroidal thickness (SFCT).

**Methods:**

This systematic review was performed on RLRL for treatment of myopia in children compared to single vision spectacles (SVS). We employed the search strategy with key terms myopia and low-level light therapy then we searched PubMed, Scopus, Cochrane, and Web of Science databases. The mean differences (MD) were used to evaluate the treatment effects. Heterogeneity was quantified using I^2^ statistics and explored by sensitivity analysis.

**Results:**

Five randomized controlled trials (RCTs) were included in our meta-analysis with a total of 833 patients, 407 in treatment group and 426 in control group. At a 3 month follow up period, pooled studies show a statistical difference in AL between RLRL and SVS group (MD = -0.16; 95% CI [-0.19, -0.12], SER (MD = 0.33; 95% CI [0.27, 0.38]), and SFCT (MD = 43.65; 95% CI [23.72, 45.58]). At a 6 month follow up period, pooled studies show a statistical difference in AL between RLRL and SVS group (MD = -0.21; 95% CI [-0.28, -0.15]), SER (MD = 0.46; 95% CI [0.26, 0.65]), and SFCT (MD = 25.07; 95% CI [18.18, 31.95]). At a 12 month follow up period, pooled studies show a statistical difference in AL between RLRL and SVS group (MD = -0.31; 95% CI [-0.42, -0.19]) and SER (MD = 0.63; 95% CI [0.52, 0.73]).

**Conclusion:**

This is the first systematic review and meta-analysis investigating only RCTs evidence supporting the efficacy of 650 nm RLRL for myopia control in the short term of 3, 6, and 12 months follow up. The present review revealed the clinical significance of RLRL as a new alternative treatment for myopia control with good user acceptability and no documented functional or structural damage. However, the effect of long-term RLRL treatment and the rebound effect after cessation require further investigations.

**Supplementary Information:**

The online version contains supplementary material available at 10.1186/s12886-024-03337-5.

## Introduction

Myopia is the most prevalent form of refractive error [1] which has a major negative impact on visual function and causes blurring of vision. It is estimated that the number of myopic patients will be 4.7 billion in the world by 2050 [2, 3]. Myopia prevalence in children aged 6 to 8 years increased 1.4–3 times during COVID-19's social isolation [4]. High myopia is linked to a significant risk of disorders that permanently impair vision, such as myopic maculopathy, glaucoma, staphyloma, and retinal detachment [5–7]. As a result myopia is a significant public health issue, and there is an urgent need for a strategy to stop its progression.

Myopia is most frequently characterized by an increase in the axial length of the eyeball [8] and can be brought on by both environmental [9] and hereditary causes [10, 11]. The threshold for myopia progression is a spherical equivalent refraction (SER) of -1.00 diopters (D) or less is [12]. The primary characteristic of high myopia-related irreversible vision loss is choroidal thinning [13]. Research on both animals and human indicates that the choroid is crucial for controlling the development of the eye's refractive system [14, 15]. As potential indicators of treatment response, axial length (AL), spherical equivalent refraction (SER), and macular choroidal thickness (mCT) have all been identified [16].

Several techniques have already been proven to be efficient preventative elements for the growth of myopia in children. Increased exposure to bright light outdoors [3, 17] is used with myopia. Increased time outdoors has been shown to prevent or delay myopia onset in several studies. Furthermore, increased outdoor time has been shown to have a protective effect on the cumulative incidence rate of myopia in children enrolled in randomized clinical trials in China and Taiwan. On the other hand, increased near-work time and reduced outdoor activities have been suggested to be at the origin of the increased myopia prevalence in older children [18]. A previous review mentioned that several studies conducted in various countries concluded that greater average daily light exposure is associated with a reduced axial elongation during childhood. For example, a study cluster-randomized intervention-controlled trial showed that exposure to outdoor light leads to less myopic shifts, reduced axial elongation and a 54% lower risk of myopia progression [19]. Additionally, single vision spectacle lenses (SVS), refractive procedures, and implantable collamer lens (ICL) are all frequently used to treat myopia. Additional methods such as orthokeratology, various contact lens options, peripheral defocus-modifying spectacle lenses, pharmacy therapy in the form of atropine eye drops, and orthokeratology were also used [20–23]. However, all of these methods have disadvantages being potentially difficult and harmful in administration. For example, the risk of adverse events associated with orthokeratology, such as infective keratitis, is well recognized [24]. Photophobia, among other side effects, is possible with atropine eye solutions [25]. Thus, there is a pressing need for more effective and practical treatments for halting myopia progression.

While increasing exposure to outdoor light levels can successfully be implemented through national outdoor programs, implementation remains suboptimal in some circumstances. On the other hand, the optimization of architectural lighting or development of light-therapy devices requires a holistic understanding of the benefits and side effects of light characteristics on ocular growth and neurophysiology [19]. The characteristics of wavelength, intensity, and other variables all have an impact on how light affects the refractive power of lenses [26]. According to research, red light has been shown to be more efficient than other wavelengths in slowing the progression of myopia in rhesus monkeys [27]. Studies have shown that repeated low-intensity red light (RLRL) can slow the progression of myopia in children [12, 25, 28] Laser therapy induces various molecular, cellular, and tissue effects [29]. Differentiating from high-power laser therapy, RLRL utilizes low doses of red and near-infrared light, with an output ranged from 1 to 500 mW, and a wavelength ranging from 600 to 1100 nm. As a result, it is described as a sort of phototherapy that emits low energy to cause tissue response [30].

Clinical studies that compared RLRL to single-vision glasses over a six-month period discovered that it significantly delayed the development of myopia and axial elongation (SVS) [28]. Several randomized clinical trials including children showed that RLRL therapy was more effective than SVS in reducing myopia, and no structural or functional impairment was detected [12]. To accurately assess the effects, it was necessary to do a meta-analysis collectively with RCT which is much better than using non-RCT. Potential biases are likely to be greater for non-randomized studies compared with randomized trials when evaluating the effects of interventions. Additionally, randomization provides a rigorous tool to examine cause-effect relationships between an intervention and outcome. Therefore, we conducted this systematic review to determine the potential benefits of RLRL treatment for childhood myopia. Our research objectives include understanding how RLRL influences the development of myopia, spherical equivalent refraction (SER), axial length (AL), and sub foveal choroidal thickness (SFCT).

## Materials and methods

This review followed the guidance of the Preferred Reporting Items for Systematic Reviews and Meta-Analyses (PRISMA) 2020 statement [31]. The filled checklist is attached in the supplementary data (Additional file 1). This systematic review was registered in PROSPERO (registration ID: CRD42023410702).

### Search strategy

A systematic review of the published literature was performed on Repeated Low-Level Red Light (RLRL) for treatment of myopia in Children. The Population-Intervention-Comparison-Outcome (PICO) question was formulated as follows: Do myopic children (P) treated with Repeated Low-level red-light therapy in addition to single vision spectacles (I) compared with Singles vision spectacles (C) have a slowed down progression of myopia with no damage or serious adverse effects? (O) We employed the search strategy attached to the supplementary data (Additional file 2): we searched PubMed, Scopus, Cochrane, and Web of Science databases. The bibliographies of all relevant articles were checked for additional articles that were not identified in our search.

### Eligibility criteria and study selection

#### Inclusion criteria

The relevant articles that met the following criteria were selected for inclusion:

(1) randomized control trials (RCTs) (2) patients with myopia younger than 16 years old, with cycloplegic spherical equivalent refraction (SER) of at least—0.50 diopter (D), (3) with the use of RLRL in addition to single vision spectacles compared to single vision spectacles only, (4) studies reported at least one of the outcomes of interest: Axial length (mm), Spherical equivalent refraction (SER) and Subfoveal choroid thickness (SFChT) with minimum 3 months follow up.

#### Exclusion criteria

Exclusion criteria included: Studies not meeting inclusion criteria, animal studies, In vitro studies, case reports, case series, non-English articles.

The duplicate records were automatically removed via the EndNote X9 computer program by (A.S) and (M.Y). Then, the remaining citations were imported to the Rayyan web application [32], which is a free web and mobile app, that helps expedite the initial screening of abstracts and titles using a process of semi-automation while incorporating a high level of usability. Thus, we used it for screening eligible studies for our systematic reviews. (R.F), (H.F) and (M.R) independently screened the citations for eligibility based on our inclusion and exclusion criteria, and they were blinded to decisions until the end of the screening process. Any conflicts that arose about the inclusion of the studies were first solved by discussion between them. (A.S) and (M.Y) were considered referees if there were unresolved discussions.

### Data extraction

For the included studies, a group of the authors extracted the data and exported the collected data into excel sheet for the following characteristics: First: Summary sheet (H.F), (R.F) and (M.R) extracted the following characteristics: Study design, location and number of center(s), sample size, follow up duration, study arms (treatment and control), patients main inclusion criteria, description of RLRL used and study conclusion. (A.S) then reviewed the final data extracted.

Second: Baseline sheet (M.R) and (A.M) extracted the following baseline characteristics of the participants: Number of patients in each arm, age, Sex (M/F), Uncorrected visual acuity, Axial length (mm), Spherical equivalent refraction and Subfoveal choroidal thickness. (A.S) then reviewed the final data extracted.

Third: Outcome sheet (A.S) and (M.Y) extracted the following outcome characteristics of the participants: Axial length (mm), Spherical equivalent refraction and Subfoveal choroid thickness.

### Risk of bias assessments of eligible studies

Using the National Institutes of Health (NIH) quality assessment tools for controlled intervention study [33], (M.R) and (A.M) independently assessed the risk of bias in the included studies, with all discrepancies being resolved in a discussion in the presence of (A.S) and (M.Y). The NIH tool consisted of 14 dichotomous (yes/no) questions. If the answer to any question was "No", then there is a potential of bias in a study.

### Data analysis

Baseline characteristics were summarized for each study and reported as mean ± standard-deviation (SD) for continuous variables, and number (%) for categorical variables. Some of the data of baseline or outcomes were presented in a study [34] as median and interquartile range (IQR). Thus, we converted these data to be presented as mean ± SD using an equation on excel sheet [35].

Standard deviations of mean changes from baseline are specified as frequently missing outcome data [36] and problems in conducting a meta-analysis without missing SDs are explained by prior systematic reviews [37, 38]. A formula was established as follows to calculate missing SDs: [39, 40]

$$SDchanged=\sqrt{{SD}^{2}baseline+{SD}^{2}final-\left(2\times r\times SDbaseline\times SDfinal\right)}$$

SD final corresponds to the SD of the post-test, SD baseline denotes the SD of the pre-test, and SD change denotes the SD of the mean changes from baseline. The r denotes the correlations between the baseline and final measurements; this correlation value is typically not presented in studies. For instance, among the included studies for this systematic review, we determined a study's coefficient (r) from its data file, and then we used the aforementioned equation to determine the missed SD change [41]. We used an equation to transform the confidence intervals (CI) from two additional included studies [12, 42] into standard deviation [43].

The I^2^ statistic, which shows the percentage of the variability in effect estimates caused by heterogeneity rather than chance [44], was used to determine the level of heterogeneity and to quantify the heterogeneity of a meta-analysis. Low (25%), moderate (50%) and high (75%) I^2^ results were assigned [45]. A meta-analysis was carried out for a continuous data, inverse variance, fixed-effect model [44] and a 95% confidence interval (95% CI) when statistical heterogeneity was absent (*p* > 0.05 in the Chi^2^ statistics). However, a meta-analysis was carried out using a more cautious random effect model for continuous data, inverse variance, and a 95% CI when statistical heterogeneity was detected [46]. Sensitivity analyses were performed by sequentially removing individual studies to determine whether each resulted in a substantial change in the magnitude or direction of the pooled estimates and heterogeneity. When the excluded study substantially changed I2 value, it is reported in the results. Meta-analyses of this review were conducted via the Cochrane Collaboration Review Manager (RevMan) software, v.5.4.1 for Windows.

## Results

### Literature search results

**Fig. 1:**
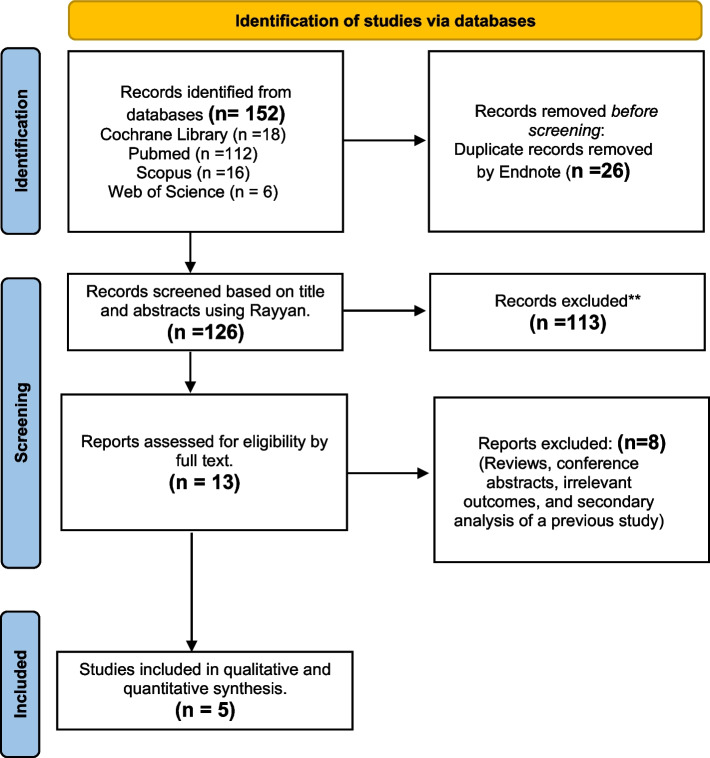
PRISMA flowchart of paper selection. PRISMA Flowchart outlining the search strategy and details on the studies finally included in the meta-analysis

Following a specific search strategy for each database. The search yielded 152 studies, after removing duplication 126 studies remain. Full-text screening results in five studies [12, 28, 34, 41, 42] available for data extraction and analysis after exclusion of irrelevant studies. PRISMA flow diagrams show the search results (Fig. 1).

### Characteristics of the included studies

Five studies were included in our mete-analysis with a total of 833 patients, 407 in treatment group and 426 in control group. These studies were published in the period from 2021 to 2023 with only two multi-center studies.

All the included studies are randomized controlled trials (RCTs). (Additional file 3) and Table 1 present the summary and the baseline characteristic of the included studies respectively. The mean age of the patients is about 10 years ranged from (7–13) years with no statistically significant difference in baseline characteristics for included children. All children in the treatment group were subjected to repeated low repeated low-level red-light (RLRL) in addition to single-vision spectacle (SVS) compared with SVS only in control group. Patients in control group didn't undergo any intervention except in Dong 2023 where the patients subjected to a sham device.

**Table 1:** Baseline characteristics of the patients

Study ID	Arm	Patients	AGE, Mean ± SD	SEX (Male/Female)	Axial length (mm), Mean ± SD	Spherical equivalent refraction(D), Mean ± SD	Subfoveal choroid thickness (μm), Mean ± SD
Xiong 2021 [27]	Treatment	74	10.22 ± 2.38	40/ 34	25.07 ± 1.15	− 3.39 ± 2.17	288.61 ± 59.59
	Control	74	10.33 ± 2.03	40 /34	25.07 ± 0.87	− 3.32 ± 1.36	286.81 ± 63.67
Jiang 2022 [12]	Treatment	119	10.46 ± 3.75	57/62	24.54 ± 0.67	- 2.49 ± 0.92	NA
	Control	145	10.53 ± 3.66	73/72	24.62 ± 0.86	- 2.67 ± 1.06	NA
Tian 2022 [33]	Treatment	112	9.66 ± (1.65)	55/57	24.31 ± 0.92	-2.17 ± 1.50	295 ± 82.98
	Control	112	9.47 ± (1.59)	57/55	24.20 ± 0.85	-2 ± 1.13	297.33 ± 81.11
Chen 2022 [40]	Treatment	46	9.00 ± (1.90)	27/19	24.62 ± 0.97	- 2.54 ± 1.04	259.00 ± 51.46
	Control	40	8.98 ± (1.92)	25/15	24.57 ± 0.76	- 2.29 ± 0.77	273.08 ± 54.37
Dong 2023 [41]	Treatment	56	10.3 ± (2.07)	26/30	24.7 ± 1.04	- 3.13 ± 1.91	NA
	Control	55	9.86 ± (1.41)	30/26	24.6 ± 0.96	- 2.82 ± 1.86	NA

### Quality assessments

**Fig. 2:**
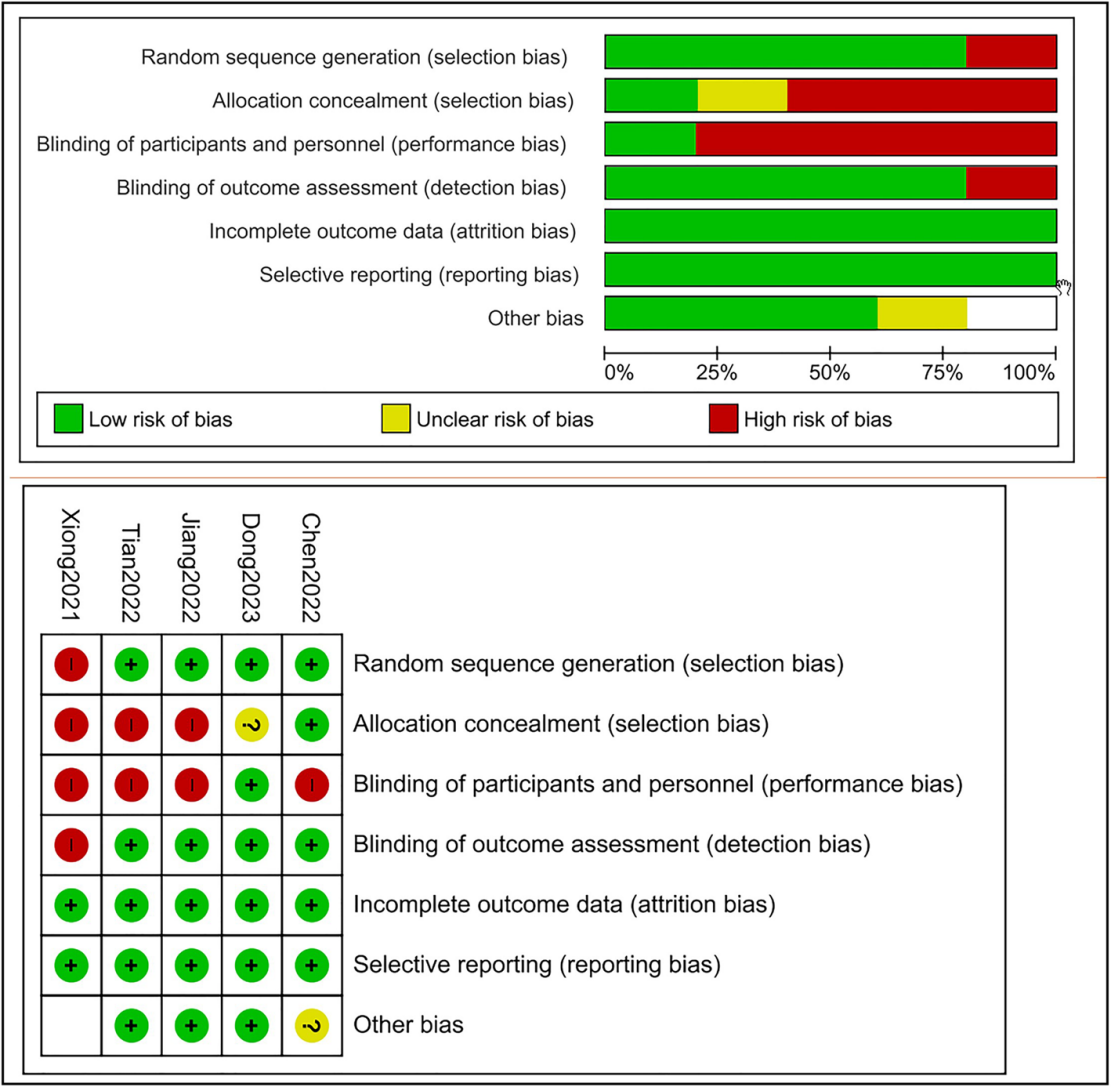
Summary of risk of bias assessment. Shows the risk of bias assessment for each trial using the Cochrane risk of bias tool. Green-colored symbol corresponds to low risk of bias, yellow corresponds to unclear risk of bias, and red corresponds to high risk of bias

According to NIH quality assessment tool, four studies have low risk of bias with a good quality and a score more than 10 except Xiong 2021 which is a low-quality study with high risk of bias. This study didn't provide sufficient details about its methodology. Most of the studies were single blinded, the patients and ophthalmologists knew the treatment group from the controlled group, and this may be due to the nature of the laser intervention; however, outcome assessors including technicians, optometrists, and statisticians were masked to the treatment allocation. On the other hand, one included study was double blinded as it used the Sham device (Dong 2023). Furthermore, Xiong 2021 which didn't report the masking. Figure 2 shows risk of bias summary and the details of the studies quality are attached in the supplementary files. (Additional file 4).

### Statistical analysis

We used mean change and standard deviation (SD) to compare results between treatment and control group. Most studies recorded data as a mean change and SD.

### Outcomes

We analyzed the difference in the three main parameters: Axial length. Spherical equivalent refraction and macular choroidal thickness during different follow up periods.

#### Axial length

**Fig. 3:**
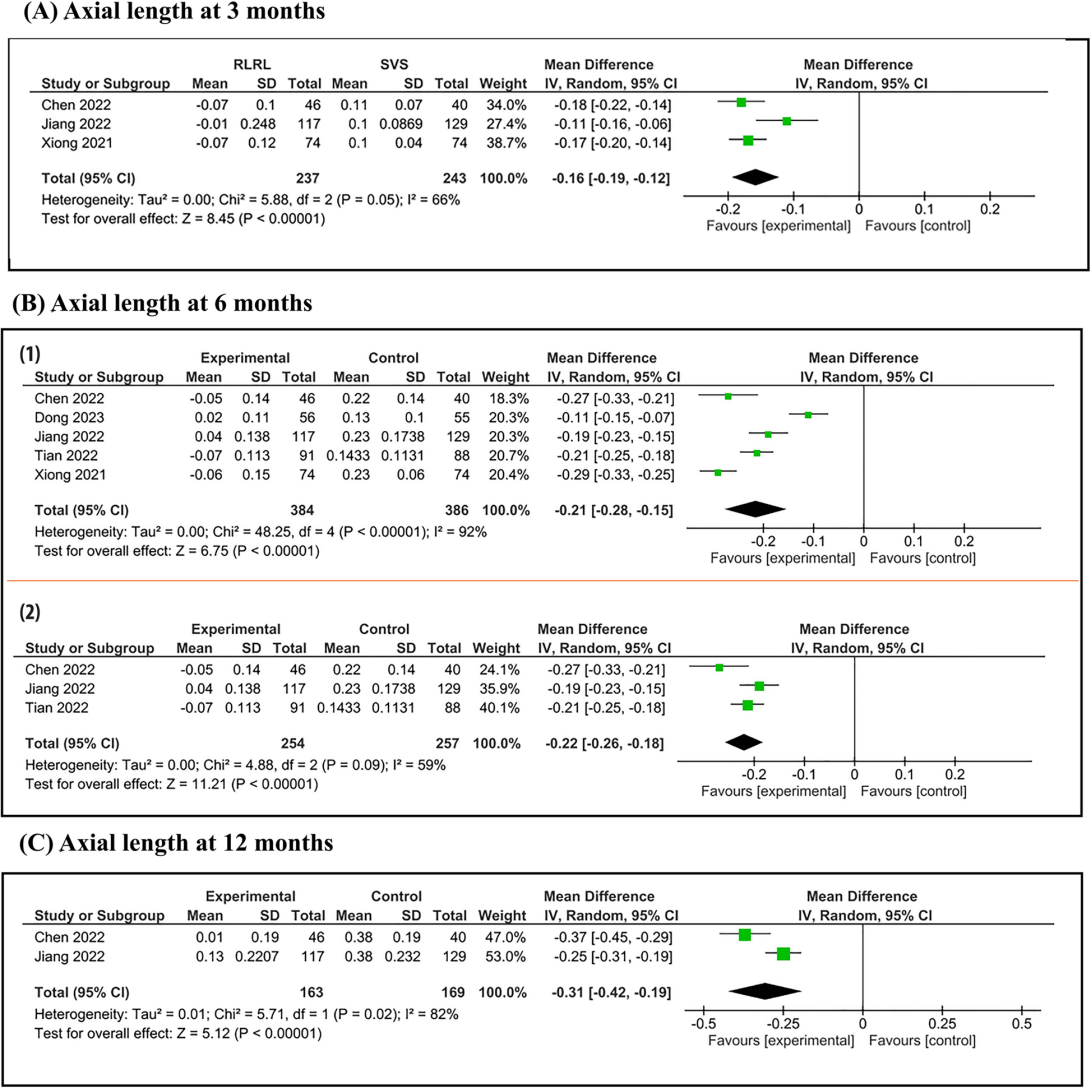
Forest plot of change in AL. Forest plot of change in AL with RLRL and single vision lens groups: (A) 3 months, (B) 6 months (1) before and (2) after sensitivity analysis, and (C) 12 months follow up interval

Axial length analysis was conducted at 3, 6 and 12 months. Figure 3 shows that:

At a 3 month follow up period, pooled studies show a statistical difference in axial length between RLRL and SVS group (MD = -0.16; 95% CI [-0.19, -0.12]). Pooled results were heterogenous (I^2^ = 66%, *P* = 0.05).

At a 6 month follow up period, there were 5 studies that reported the change in axial length from the baseline. Pooled studies show a statistical difference in axial length between RLRL and SVS group (MD = -0.21; 95% CI [-0.28, -0.15]). Pooled results were heterogeneous (I^2^ = 92%, *P* < 0.00001).

At a 12 month follow up period, pooled studies show a statistical difference in axial length between RLRL and SVS group (MD = -0.31; 95% CI [-0.42, -0.19]). Pooled results were heterogeneous (I^2^ = 82%, *P* = 0.02).

#### Spherical equivalent refraction

**Fig. 4:**
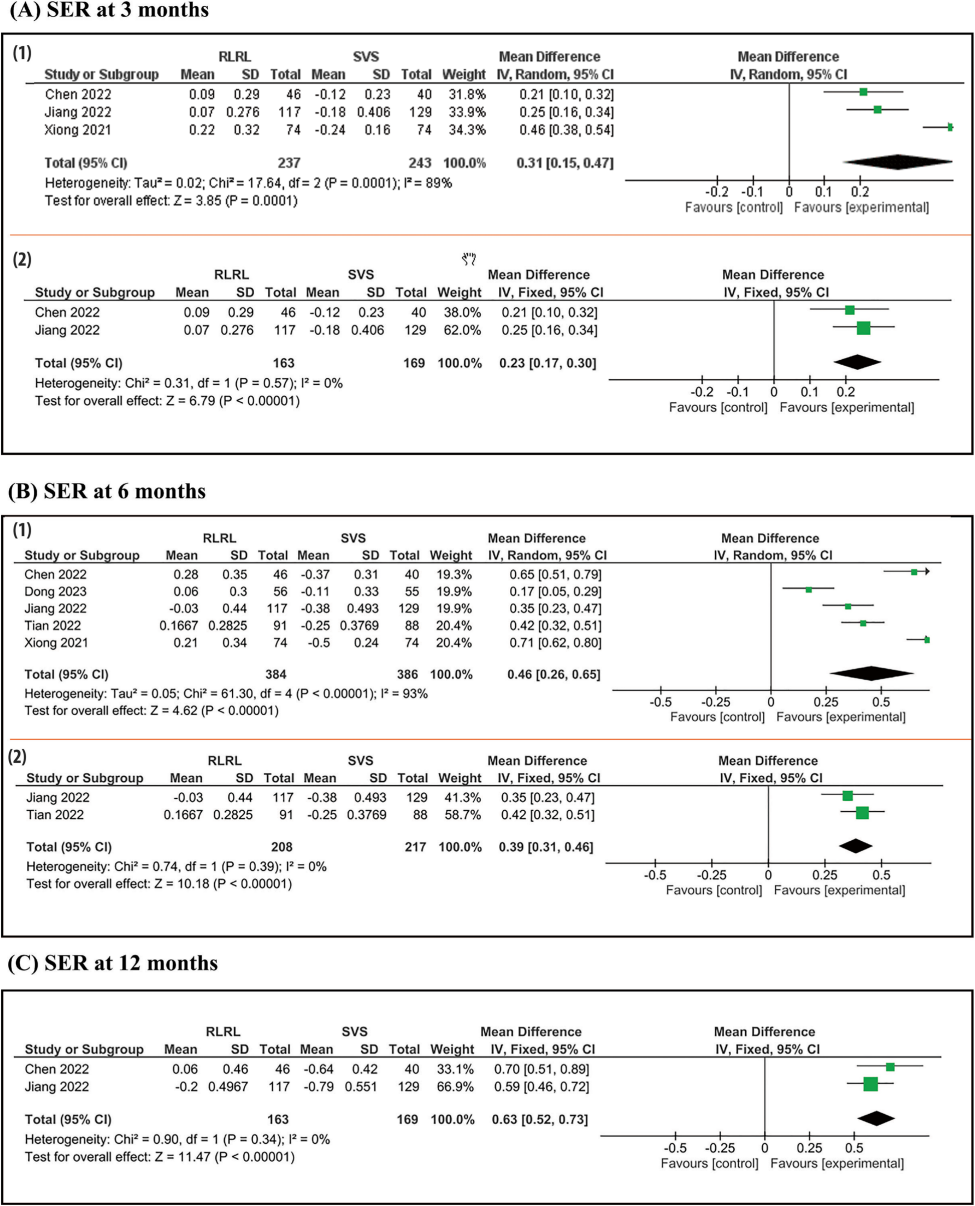
Forest plot of change in SER. Forest plot of change in SER with RLRL and single vision lens groups: (A) 3 months (1) before and (2) after sensitivity analysis (B) 6 months (1) before and (2) after sensitivity analysis, and (C) 12 months follow up interval

SER analysis was conducted at 3 and 6 and 12 months. Figure 4 shows that:

- At a 3 month follow up period, we analyzed 3 studies, pooled studies show a statistical difference in SER between RLRL and SVS group (MD = 0.33; 95% CI [0.27, 0.38]). Pooled results were heterogeneous (I^2^ = 89%, *P* = 0.0001).
- At a 6 month follow up period, we analyzed 5 studies, pooled studies show a statistical difference in SER between RLRL and SVS group (MD = 0.46; 95% CI [0.26, 0.65]). Pooled results were heterogeneous (I^2^ = 93%, *P* < 0.00001).
- At a 12 month follow up period, pooled studies show a statistical difference in SER between RLRL and SVS group (MD = 0.63; 95% CI [0.52, 0.73]). Pooled results were homogeneous (I^2^ = 0%, *P* = 0.34).

#### Macular choroidal thickness

**Fig. 5:**
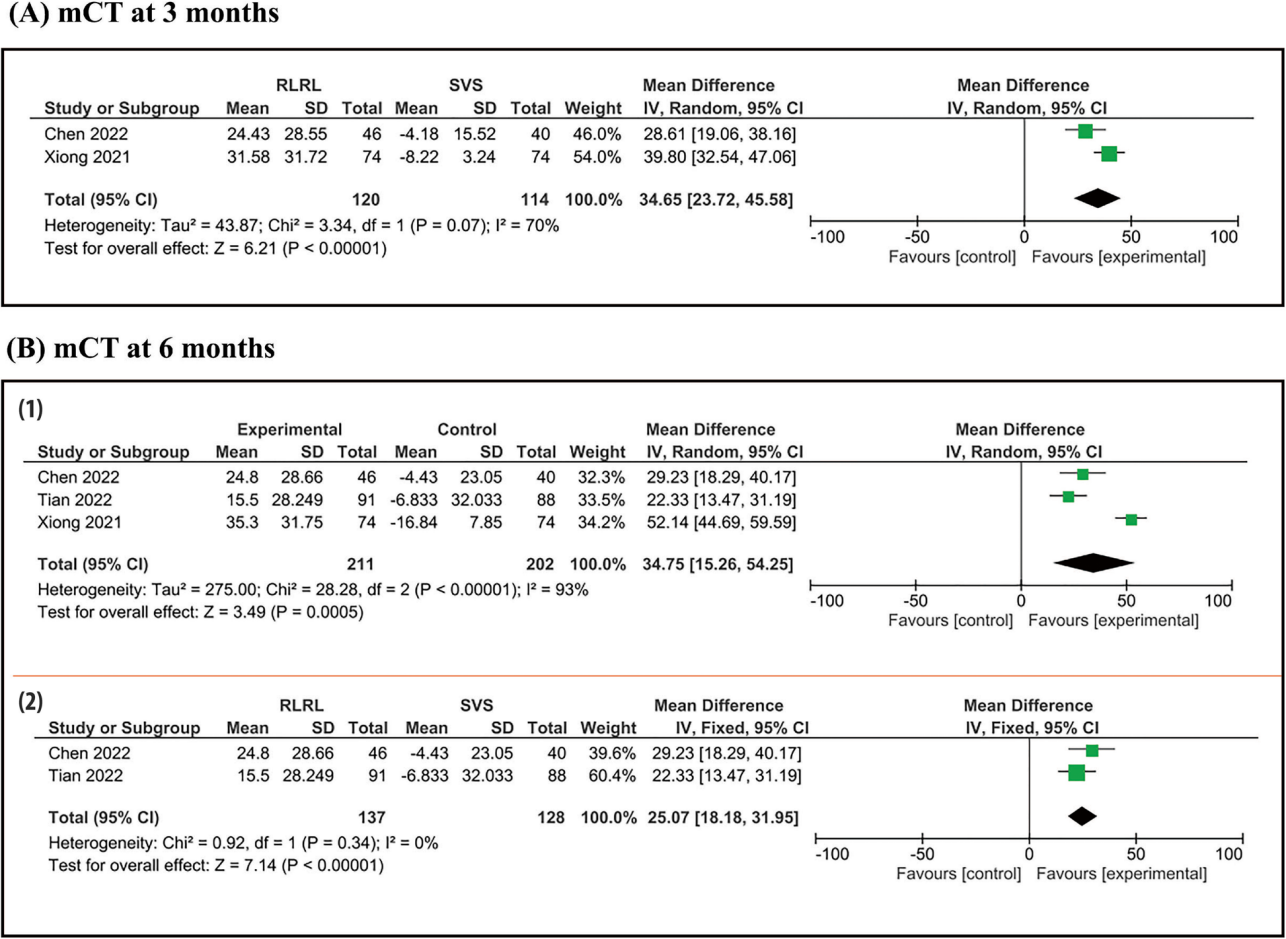
Forest plot of change in SFCT. Forest plot of change in SFCT with RLRL and single vision lens groups: (A) 3 months and (B) 6 months (1) before and (2) after sensitivity analysis

mCT analysis was conducted at 3 and 6 months. Figure 5 shows that:

- At a 3-month, pooled studies show a statistically difference in macular choroidal thickness between RLRL and SVS group (MD = 34.65; 95% CI [23.72, 45.58]). Pooled results were heterogenous (I^2^ = 70%, *P* = 0.07).
- At a 6-month, pooled studies show a statistically difference in macular choroidal thickness between RLRL and SVS group (MD = 34.75; 95% CI [15.26, 54.25]). Pooled results were heterogenous (I^2^ = 93%, *P* < 0.00001).

### Sensitivity analysis and heterogeneity

Meta-analysis showed heterogeneity in some studies, to overcome this heterogeneity we used random effect model instead of fixed effect model. There was no sufficient data to do subgroup analysis so we couldn't overcome the heterogeneity. Finally, we used leave one out method in trying to overcome the heterogeneity, however, it still presents in some outcomes.

#### Axial length

At a 6 month follow up period, a significant heterogeneity was noted when pooling all the studies, so we made a sensitivity analysis by removing two studies Dong 2023 and Xiong 2021 and there was still a statistically significant difference and the heterogenicity decreased (I^2^ = 59%, *P* = 0.09). (Fig. 3-b-2).

At a 12 month follow up period, pooled results were heterogeneous (I^2^ = 82%, *P* = 0.02) and we couldn't solve this heterogeneity by sensitivity or subgroup analysis due to limited number of studies.

### Spherical equivalent refraction

At a 3 month follow up period, pooled results were heterogeneous (I^2^ = 89%, *P* = 0.0001). After sensitivity analysis, we removed Xiong 2021 and there was still a statistically significant difference with no heterogenicity (I^2^ = 0%, *P* = 0.57). (Fig. 4-a-2).

At a 6 month follow up period, pooled results were heterogeneous (I^2^ = 93%, *P* < 0.00001). After sensitivity analysis, we removed Dong 2023 and Xiong 2021 and there was no heterogenicity (I^2^ = 0%, *P* = 0.39). (Fig. 4-b-2).

### Macular choroidal thickness

At a 6 month follow up period, pooled results were heterogeneous (I^2^ = 93%, *P* < 0.00001). After sensitivity analysis, we Xiong 2021 and there was no heterogenicity (I^2^ = 0%, *P* = 0.34). (Fig. 5-b-2).

At a 3-month, pooled results were heterogenous (I^2^ = 70%, *P* = 0.07) and we couldn't solve this heterogeneity by sensitivity or subgroup analysis due to limited number of studies.

## Discussion

This is a systematic review specifically conducted to investigate whether repeated low-level red light (RLRL) therapy can control the progression of myopia in children. The efficacy of the RLRL can introduce an alternative which seems at least competitive with other treatment methods. Our results showed that RLRL can better control axial length (AL) elongation, spherical equivalence refraction (SER), and subfoveal choroidal thickness (SFCT) better than single vision spectacles (SVS) within 3, 6, 12 months when compared to wearing single-vision glasses. All the outcomes were reported at 6 months follow up; however, AL was reported in 3 months and SER was reported in 12 months additionally. AL was defined as the distance from the corneal vertex to the retinal pigment epithelium, representing myopia. AL was improved compared to baseline values at 3 month follow up and 6 month follow up periods. SER was improved after using RLRL compared to baseline at 3 months follow up, 6 months follow up, and 12 months follow up. SFCT was increased compared to baseline at 6 months follow up.

The heterogeneity was significantly high when pooling all studies. To investigate the heterogeneity, we couldn’t do subgroup analysis due to limited data. However, we excluded from the quantitative analysis the studies that were responsible for heterogeneity by conducting sensitivity analysis. Sensitivity analysis showed changes in the heterogeneity, however, there was still a statistically significant difference. The studies removed by leave one out may have some limitations due to the design, sample size, or the procedures. For example, we removed the data of Xiong 2021 due to significant heterogeneity detected in all outcomes except AL of 3 months. This study was of low quality according to the quality assessments tool which may have resulted from the methods of randomization and allocation. Additionally, the laser power was very different in this paper compared to other papers (Additional file 3). Another example, we removed the data of Dong 2023 also due to significant heterogeneity detected in all outcomes without exception. The heterogeneity of this study could be related to using sham device that used 10% of the original device’s power compared with the SVS control group which improved the cases to an extent. Sensitivity analysis showed solving of some heterogeneity however, the significant differences were still found between the two groups.

Our results are consistent with a recently published systematic review and meta-analysis (Tang et al. 2023) [47] discussing the RLRL therapy effectiveness. Tang et al. 2023 concluded that the weighted mean difference (WMD) for myopia progression between RLRL and the control group was 0.68 D per 6 months for SER change, -0.35 mm per 6 months for AL elongation and 36.04 μm per 6 months for SFChT change. However, this recently published study included non-randomized controlled trials as cohort and post-hoc analysis studies that may affected the heterogeneity and validity of the results.

No severe adverse events, such as scotoma that occurred during the trial linked to the intervention or sudden vision loss by 2 lines happening over the course of a few seconds or minutes to a few days, were reported in any of the included studies. This is consistent with Tang et al. 2023 which reported that none of the studies in their meta-analysis reported vison-threatening events and no structural damage was observed in the photosensory layers. However, a recent case report of 12-year-old female mentioned the possibility of retinal damage after RLRL exposure [48]. Based on the findings of the short-term follow-up, RLRL intervention has a promising control impact on myopia. Contrarily, the risk of adverse events associated with orthokeratology, such as infective keratitis, is well recognized [24]. Photophobia, among other side effects, is possible with atropine eye solutions [49–51].

Currently, much empirical evidence suggests that oxidative stress and inflammation may be responsible for the altered regulatory pathways in myopia, and that oxidative damage of hypoxic myopia can change how nitric oxide and dopamine are neuromodulated during eye development [52, 53]. It may be feasible to protect patients from the effects of oxidative stress and reduce inflammation associated with myopia by investigating the potential mechanisms underlying the inhibitory effects of RLRL treatment [54]. In both animal research [55] and clinical studies [28, 56], RLRL has the greatest effects on the nitric oxide system and reduces the intensity of oxidative stress. By inhibiting inflammatory cytokines like interleukin-1 (IL-1) and tumor necrosis factor-α, RLRL may lower their amounts [57]. Additionally, severe myopia may substantially raise IL-1 and IL-6 levels, which may be related to the myopic control mechanism [58, 59]. According to a generally accepted theory, bright light causes the retina to produce and release more dopamine [60]. In the development of refractive eyes, dopamine functions as a termination signal [61]. 

The choroid has a variety of functions, including nourishing the retina [62] and can affect the refractive state [63, 64]. Furthermore, the choroid has a crucial role in relaying signals derived from the retina to the sclera, further altering the synthesis of scleral extracellular matrix and changing the ocular size, resulting in refractive changes that have a vital function in the etiology of myopia [65, 66]. Additionally, the release of nitric oxide from the retina or choroid by the dopamine in the retina may cause choroidal thickening and inhibit ocular growth [67, 68]. After 650 nm RLRL treatment, previous research found a significant increase in choroidal thickness. Similarly, Gawne et al. [69] noted a refractive shift brought on by a decrease in vitreous chamber depth and a rise in choroid thickness. Two of the included studies [12, 41] hypothesized that RLRL may control myopia by increasing choroidal blood flow and reducing oxidative stress and inflammation [70, 71], and thus ameliorating scleral hypoxia.

Other presently available non-invasive interventions, such as more outdoor exercise, orthokeratology, and atropine eye drops, may not have the same myopia-controlling effects as 650 nm RLRL. Over the past few years, outdoor time has drawn a lot of focus. According to He et al. [72], spending more time outside did reduce the progression of myopia compared to the control group. Despite an extra 40 min of outdoor activity being added to each school day, children still on average underwent a -1.42 D myopic shift and a 0.95 mm elongation of AL during the 3-year follow-up. Outdoor exercise, in contrast, appeared to be less effective than 650 nm RLRL, let alone the difficulty of implementing an extra 40 min of outdoor exercise given the demands of students' academic work.

In terms of clinical implication from this review, repeated low-level red-light intervention was needed twice daily, 3 min per session, five days per week which is the same procedure for treating amblyopia. The investigators of one of the included studies (Jiang 2022) gave parents the device to allow this daily treatment schedule so they could carry out this treatment at home. Users must log in to the system using a specified username and password given to start treatment because the device is connected to the internet. The research coordinator will be able to watch, record, and keep track of treatment adherence when using devices. Jiang et al.'s study [12] showed that greater treatment compliance greatly increased treatment efficacy. This significant dose response effect may further support the effectiveness of RLRL in controlling myopia, but it also emphasizes the importance of putting up a suitable incentive system to motivate kids to use the device and to maximize treatment efficacy. This potent dose–response relationship also suggests that treatment efficacy may be enhanced by increasing the treatment duration from 3 min to an extended treatment time per session.

However, there were some limitations in our review. First, the number of studies included in this meta-analysis was limited. Because of the limited number of included studies and hence limited statistical power, publication bias test was not performed in our review according to Begg’s and Egger’s recommendation [73]. Second, considering the significant methodological heterogeneity among included studies, we couldn't conduct subgroup analysis due to limited data. Third, the follow up periods were short to evaluate the potential dose–response relationship and the optimal power intensity of the RLRL treatment for myopia control. Fourth, all the published studies were limited to centers in China. Thus, we recommend further studies with long-term follow-up involving various centers in different countries to confirm our findings. Furthermore, we do not know whether there is a rebound effect like atropine eye drops once treatment is stopped, which is a knowledge gap for future study.

## Conclusion

In summary, this is the first systematic review and meta-analysis investigating only RCTs evidence supporting the efficacy of 650 nm RLRL for myopia control in the short term of 3, 6, and 12 months follow up. The present review revealed the clinical significance of RLRL for myopia control in terms of AL, SER, and SFCT. It has slowed down and reversed the myopia progression in a large proportion of children. RLRL therapy is an effective new alternative treatment for myopia control with good user acceptability and no documented functional or structural damage. However, the effect of long-term RLRL treatment and the rebound effect after cessation require further investigation.

### Supplementary Information

**Additional file 1.** PRISMA 2020 Checklist.

**Additional file 2.** Search strategy.

**Additional file 3.** Table showing summary sheet of characteristic of the included studies.

**Additional file 4.** Table showing risk of bias summary and the details of the studies quality.

## Data Availability

The datasets used and/or analyzed during the current study are available from the corresponding author on reasonable request.

## Abbreviations

RLRL: Repeated Low-Level Red Light
SVS: Singe vision spectacles
AL: Axial length
SER: Spherical equivalent refraction
SFCT: Sub foveal choroidal thickness
MD: Mean difference
RCTs: Randomized controlled trials
D: Diopters
mCT: Macular choroidal thickness
ICL: Implantable collamer lens
PRISMA: Preferred Reporting Items for Systematic Reviews and Meta-Analyses
PICO: The Population-Intervention-Comparison-Outcome
Sex (M/F): Male/Female
NIH: National Institutes of Health
SD: Standard-deviation
IQR: Interquartile range
r: Denotes the correlations between the baseline and final measurements
CI: Confidence intervals
IL-1: Interleukin-1
NMA: Network meta-analysis
