# Supplementary material for: Efficacy of Repeated Low-Level Red Light (RLRL) therapy on myopia outcomes in children: a systematic review and meta-analysis
Source: BMC Ophthalmol. 2024 Feb 20;24:78. doi: 10.1186/s12886-024-03337-5 (PMC10877869; doi:10.1186/s12886-024-03337-5)
Supplement: Supplementary file 2 — Additional file 2. Search strategy. [file 12886_2024_3337_MOESM2_ESM.docx]

**Search strategy:**

Pubmed 112
(((((((Myopia) OR (Myopias)) OR (Nearsightedness)) OR (Nearsightednesses)) AND (Low-Level Red Light)) OR (Repeated Low-Level Red Light)) OR (RLRL)

Scopus 16 & WOS 6 & Cochrane 18

(Myopia OR Myopias OR Nearsightedness OR Nearsightednesses) AND (Low-Level Red Light OR Repeated Low-Level Red Light OR RLRL)
